# The effects of adolescence sports and exercise on adulthood leisure-time physical activity in educational groups

**DOI:** 10.1186/1479-5868-7-27

**Published:** 2010-04-12

**Authors:** Tomi E Mäkinen, Katja Borodulin, Tuija H Tammelin, Ossi Rahkonen, Tiina Laatikainen, Ritva Prättälä

**Affiliations:** 1 National Institute for Health and Welfare (THL), Helsinki, Finland; 2 Finnish Institute of Occupational Health, Oulu, Finland; 3 LIKES Research Center, Jyväskylä, Finland; 4 University of Helsinki, Department of Public Health, Helsinki, Finland

## Abstract

**Background:**

Physical inactivity has become a major public health problem and clear educational differences in physical activity have been reported across Europe and USA. The origins of adulthood physical activity are suggested to be in childhood and adolescence physical activity. Hardly any studies have, however, examined if the educational differences in physical activity might also be due to educational differences in early experiences in physical activity. Thus, our aim was to examine how competitive sports in youth, and exercise in late adolescence, and opinions on physical education (PE) in childhood determined adulthood leisure-time physical activity (LTPA) in different educational groups.

**Methods:**

We used cross-sectional population-based National FINRISK 2002 data for 1918 men and 2490 women aged 25 to 64 years. Competitive sports in youth, exercise in late adolescence, and opinions on PE in childhood were assessed retrospectively via self-reports. Adulthood LTPA was collected with 12-month recall. In 2008, we calculated structural equation models including latent variables among the low- (<12 years) and high- (≥12 years) educated.

**Results:**

Men more often than women reported that their experience of PE was interesting and pleasant as well as having learned useful skills during PE classes. Men, compared to women, had also been more active in the three selected competitive sports in youth and exercised in late adolescence. Participation in competitive sports in youth among the low-educated and exercise in late adolescence among the high-educated had a direct effect on adulthood LTPA. Among the low-educated, opinions on PE in childhood had an indirect effect on adulthood LTPA through participation in competitive sports in youth whereas among the high-educated, the indirect effect went through exercise in late adolescence. The effects were mainly similar between genders.

**Conclusions:**

Our study answers to a strong need to assess the determinants of leisure-time physical activity to promote physical activity in low-educated individuals. The pathways of physical activity from childhood to adulthood LTPA may be different depending on the pursued educational career. Further prospective studies are needed to confirm our results.

## Introduction

Physical inactivity has become a public health problem in many industrialized countries [1-3], with few people meeting the recommendations for physical activity [4]. Moreover, population-based studies from the USA [5] and Canada [6,7] as well as from European countries [3,7-10] show that lower-educated people report lower levels of physical activity. The low-educated people therefore also miss the beneficial health effects of regular physical activity [4,11]. A strong need exists to assess the determinants of leisure-time physical activity (LTPA) to promote physical activity in low-educated individuals.

Physical activity has been shown to decrease during the transition from youth to adulthood [12,13]. Tracking of physical activity in adolescence and from youth to adulthood have varied from low to moderate. The tracking correlations are higher when the time points of the two measurements are close [12]. Several longitudinal studies [14-17], however, suggest that physically active individuals tend to stay physically active from adolescence to adulthood. During adolescence, LTPA is more regular among those who participate in several different types of sports after school hours compared to those who participate in only one sport [18]. This diverse adolescent participation in sports and exercise--for example running, cross-country skiing, and endurance sports in men, and running, track and field, and orienteering in women--also associates to higher levels of physical activity in adulthood [12,15,19].

Prospective research shows that not only LTPA [16] but also physical education (PE) [14] explain adulthood LTPA. A nationally representative prospective study in the USA [20] showed that high participation in school physical activities, including team and individual sports, academic clubs, and PE, was associated with adulthood LTPA. In a recent prospective study [21], the average minutes of PE did not predict adulthood total physical activity or fitness. It has been shown that also high grade in PE [12] explains adulthood LTPA. In addition, physically active adolescents might progress better in their educational career than their physically inactive counterparts [14,22]. Sizable amount of evidence exists [23,24] that enjoyment of exercise, self-efficacy and value of expected outcomes have positive effects on physical activity. However, there is, to our knowledge, no information how enjoyment, pleasantness and usefulness of PE in childhood at population level could motivate to participate in sports and exercise in adolescence as well as in LTPA in adulthood. One might hypothesize that those opinions on PE in childhood could directly affect to physical activity and exercise in youth and which could indirectly affect to adulthood LTPA.

The roots of adulthood unhealthy behaviours, as well as for physical inactivity, may lay in childhood socioeconomic conditions [25], and in childhood and adolescence health behaviours [26]. Physically strenuous work during the lifespan may as well affect on willingness and ability to participate in LTPA [27]. Some qualitative studies [28,29] suggest that, among adults, determinants of physical activity might depend on the socioeconomic position. Recent studies [30,31] showed that there might be socioeconomic variation in parental support and perceived outcomes among adolescents which determine their physical activity. No studies, to our knowledge, have examined how the socioeconomic variation in childhood and adolescence physical activity might affect on adulthood physical activity. Thus, our aim was to examine how retrospective information on competitive sports in youth, exercise in late adolescence, and opinions on PE in childhood determine adulthood LTPA in low- and high-educated groups. We assumed that there might be unobserved constructs, such as socioeconomic cultural team spirit, behind participation in competitive sports in youth and opinions on PE in childhood that could vary in low- and high educated groups.

## Methods

### Study design

This population-based cross-sectional study was part of the National FINRISK 2002 study [32]. Data were collected using an area, gender, and age-group stratified random sample drawn from the population register. Approximately two-thirds of the original sample (N = 13 436) were randomized into the FINRISK 2002 physical activity sub-study (N = 9179). The participation rate was 60 percent for men and 70 percent for women. Participants aged 65 to 74 (N = 692) were excluded from the analyses for several reasons. Mainly because this age-group was examined only in two geographical study areas and thus was not a representative sample. Other reasons were their larger amount of leisure-time and health reasons for not being able to participate on physical activity. In addition, those who had given insufficient information in a 12-month self-administered recall questionnaire on LTPA (N = 830) were excluded. The final sample included 1918 men and 2490 women.

The entire study protocol followed the WHO MONICA protocol [33] and later the recommendations of the European Health Risk Monitoring Project [34]. The Hospital District of Helsinki and Uusimaa's Ethics Committee for Research in Epidemiology and Public Health approved the study protocol and the participants provided their written consent.

#### PE setting in Finland

The Finnish school system has varied between the age-cohorts. Those born between the 1930s and the 1970s had normally six years of elementary school whereas those born after the 1970s have had nine years. After elementary school, if one aims for a higher educational degree, one has to complete senior high school. Among every age cohorts, education has included compulsory PE. Although it is very hard to tract the actual PE, the amount of PE has been reduced during the 20^th ^century. Generally the more optional years of education you have the more physical activity opportunities you will have.

#### Measures

*Adulthood LTPA *was collected with a detailed 12-month self-administered recall questionnaire which has been validated in the Kuopio Ischemic Heart Diseases Risk Factor Study [35]. A trained nurse instructed participants in filling out the recall. The recall provided information on frequency, duration and intensity for 23 of the most common types of LTPA. The outcome measure, total LTPA, was metabolic equivalents multiplied by hours per week (METh/wk), where 1 MET-hour equals energy expenditure of 1kcal/kg/h at rest. The metabolic cost of each physical activity was based on the Ischemic Heart Diseases Risk Factor Study protocol [36] and other internationally accepted norms [37]. For the analyses, the LTPA was log-transformed due to its skewness.

Information on opinions on PE in childhood, participation in competitive sports in youth, and exercise in late adolescence were asked retrospectively. *Opinions on PE in childhood *included six statements on a scale of one (I entirely agree) to five (I entirely disagree) concerning participants' PE during their school years. For analyses, two items were summarized into three categories: 1 = "I disagree", 2 = "I somewhat agree", and 3 = "I entirely agree". Information on participants' *competitive sports in youth *was formed from two questions: at what age did you participate in competitive sports and in what events did you compete. Three of the most common events were used in the analyses: running, cross-country skiing, and track and field. In the analyses, those who participated in these events before the age of 15 were compared to those who participated in different events or did not participate at all in competitive sports in youth. Participants' frequency of *exercise in late adolescence *was collected with a question: How often did you exercise in your leisure-time (including jogging, cross-country skiing, cycling, swimming, walking, pole/Nordic walking, aerobics, ball games, ice hockey, etc.) at age of 15 to 24 years". Responses fell into four categories: 1 = "once a week or less often", 2 = "from two to three times a week", 3 = "from four to five times a week" and 4 = "more than five times a week".

Participant's *education *was collected in the questionnaire as years spent in full time education. For multi-group analyses, it was dichotomized into two categories: those who had not graduated from senior high school (low education, less than 12 years spent in full time education) and those who had a senior high school degree (high education, 12 years or more).

### Statistical methods

Descriptive analyses were done with STATA 9.2 [38]. Correlation matrix, means, and standard deviations (SD) for the examined variables were calculated. Characteristics of the study sample by age, gender and educational group are presented in proportions (%), and medians (ME). 25^th ^and 75^th ^percentiles are shown for median values. Chi^2 ^p-values for age, gender and educational differences were also calculated. In addition, Cronbach's alpha (α) was calculated for opinions on PE and participation in competitive sports in youth to examine internal reliability.

We applied structural equation models with latent variables to examine different types of adolescent physical activity as determinants of adulthood LTPA in educational groups. To be able to fully understand the effects of adolescence factors to adulthood LTPA, one must examine both the direct and indirect effects as well as the latent variables that cannot be strictly measured. Structural equation modelling combines a possibility to examine different patterns of causal effects and to use observed variables to measure underlying factors [39,40].

We estimated all the structural equations models with Mplus 5.2 software [39] using weighted least squares [41] due to the combination of ordinal and continuous outcomes. Commonly accepted model fit indexes and cut-off points indicating a good fit [42] were used to assess the model fit to the data: Tucker-Lewis Index (TLI) ≥ 0.95, Comparative Fit Index (CFI) ≥ 0.96, Root Mean Square Error of Approximation (RMSEA) ≤ 0.05, and Weighted Root Mean Square Residual (WRMR) ≤ 1.00. In the structural equation modeling we followed the two-step rule [40]: first the measurement models (only latent variables) were estimated and evaluated against the model's fit indexes, and, secondly, the structural part of the model was added. The latter was done sequentially by adding one variable at a time into the model and in each step the model's fit indexes were evaluated. All the associations between the examined variables were chosen based on chronological order. The results are based on the final model.

Age-group structural models were analysed to determine the possible age effect of the associations between youth and adolescent determinants and adulthood LTPA. The direct and indirect effects from the educational-group structural equation analyses are reported for men. In all of the models, the indirect effect is the product of all the direct effects between the variables of interest. The squared residuals (R^2^) for each variable describe the percentage of the variable's variance explained by other variables in the model. We present only the standardized solution, which enables a comparison of the coefficients (= B) within in the low-educated group and the high-educated group.

## Results

### Characteristics of the study sample by age, gender and education

Table 1 and Table 2 show the correlations between the examined variables. The correlations were mostly similar between women and men. The items of opinions on PE correlated strongly with each other strongly (correlation from 0.61 to 0.71) as did the items in participation on competitive sports (from 0.18 to 0.56). Education had a correlation of 0.11 to adulthood LTPA. Exercise in late adolescence had the highest correlation of 0.15 to adulthood LTPA.

**Table 1:** Correlations*, means and standard deviations (SD) for examined variables for men.

CORRELATIONS:	PE^a ^was interesting and pleasant	I learned useful PA skills in PE	Running	Cross-country skiing	Track & Field	Exercise in late adolescence	Adulthood LTPA^b ^(log-transformed)	Education (years)	Age (years)
**PE was interesting and pleasant**	1								
**I learned useful PA skills in PE**	0.61	1							
**Running**	0.16	0.15	1						
**Cross-country skiing**	0.19	0.17	0.51	1					
**Track & Field**	0.17	0.14	0.56	0.42	1				
**Exercise in late adolescence**	0.23	0.15	0.2	0.18	0.18	1			
**Adulthood LTPA (log-transformed)**	0.01	0.02	0.05	0.06	0.04	0.15	1		
**Education (years)**	-0.16	-0.1	-0.03	-0.04	0.03	0.06	0.13	1	
**Age (years)**	0.11	0.07	0.06	0.09	0.06	0.01	-0.05	-0.32	1
**MEAN:**	2.34	2.16	0.09	0.14	0.08	1.42	2.85	12.65	45
**SD:**	0.72	0.71	0.29	0.34	0.28	1.05	1.02	3.77	11.22

* All the correlations were statistically significant p ≤ 0.05

^a ^PE, physical education

^b ^LTPA, leisure-time physical activity

**Table 2:** Correlations*, means and standard deviations (SD) for examined variables for women.

CORRELATIONS:	PE^a ^was interesting and pleasant	I learned useful PA skills in PE	Running	Cross-country skiing	Track & Field	Exercise in late adolescence	Adulthood LTPA^b ^(log-transformed)	Education (years)	Age (years)
**PE was interesting and pleasant**	1								
**I learned useful PA skills in PE**	0.71	1							
**Running**	0.15	0.15	1						
**Cross-country skiing**	0.21	0.18	0.48	1					
**Track & Field**	0.12	0.13	0.5	0.37	1				
**Exercise in late adolescence**	0.23	0.23	0.11	0.11	0.09	1			
**Adulthood LTPA (log-transformed)**	-0.01	0.04	0.04	0	0.02	0.1	1		
**Education (years)**	-0.25	-0.18	-0.004	-0.07	0.01	-0.001	0.11	1	
**Age (years)**	0.19	0.12	-0.07	0.04	-0.08	0.01	0.01	-0.39	1
**MEAN:**	2.01	1.99	0.07	0.1	0.05	1.23	2.93	13.33	44.3
**SD:**	0.78	0.74	0.27	0.32	0.25	1.03	0.96	3.68	11.34

* All the correlations were statistically significant p ≤ 0.05

^a ^PE, physical education

^b ^LTPA, leisure-time physical activity

The basic characteristics of the study sample can be seen in Table 3. Men more often than women reported that their experience of PE was interesting and pleasant as well as having learned useful skills during PE classes. Men, compared to women, had been more active in the three selected competitive sports in youth and exercised in late adolescence. The low-educated, compared to their high-educated counterparts, agreed more often that PE had been interesting and pleasant and had learned useful physical activity skills in PE classes. The low-educated had been more active than the high-educated in cross-country skiing and in track and field. The low-educated, on the other hand, had engaged less often in exercise during their late adolescence than their high-educated counterparts. The high-educated participants reported more LTPA in adulthood than their low-educated counterparts.

**Table 3:** Characteristics of the participants by age, gender and educational group^a^. Values are proportions (%) or medians (Me)

	25-44 age-group	45-64 age-group	Men	Women	Low education	High education	P-value^f ^for age differences	P-value^f ^for gender differences	P-value^f ^for educational differences
**Opinions on PE^b^:**									
**a) PE was interesting and pleasant (%)**							<0.001	<0.001	<0.001
I disagree	28	19	14	31	13	29			
I somewhat agree	39	35	37	38	35	39			
I entirely agree	32	46	49	32	52	32			
**b) I learned useful physical activity skills in PE classes (%)**							<0.001	<0.001	<0.001
I disagree	27	22	18	29	18	28			
I somewhat agree	46	44	47	42	46	44			
I entirely agree	27	35	34	29	36	29			
**Participation in competitive sports in youth (%)^c^:**									
Running	8	7	9	7	8	8	0.095	0.006	0.744
Cross-country skiing	10	13	14	10	13	10	0.025	<0.001	0.006
Track and field	7	6	8	5	5	7	0.281	<0.001	0.023
**Exercise in late adolescence (%)^d^**							<0.001	<0.001	<0.001
Once a week or less	22	29	23	27	30	23			
2-3 times a week	39	32	33	37	32	37			
4-5 times a week	23	21	24	20	21	22			
>5 times a week	16	18	20	15	17	17			
**Leisure-time physical activity, METh/week, [Me^e^]**	19.7 [10;35]	18.9 [9;34]	19.1 [8;35]	19.4 [10;34]	16.3 [7;32]	20.9 [11;35]	0.014	<0.001	<0.001

^a ^The cut-off point for educational group analyses was those who had not graduated from senior high school (low education) and those who had senior high school degree (high education)

^b ^PE = Physical Education

^c ^Participants who had started competitive sports before the age of 15

^d ^Participants 's participation in exercise when they were aged 15 to 24 years

^e ^25th and 75th percentile are presented in brackets [25th percentile,75th percentile]

^f ^P-values from Chi^2 ^test

### The latent variables, direct and indirect effects in age and educational groups

The fit of the measurement model (only latent variables) to the data was good in men and women. The correlation between PE in childhood and participation in competitive sports in youth was 0.40 in men and 0.39 in women (P < 0.001). PE indicators implied a positive association to adulthood LTPA (P < 0.001), as was also the case in the three competitive sports indicators (P < 0.001). Cronbach's alpha was 0.82 for PE items and 0.72 for competitive sports items, which indicates good internal validity. In age-group analyses (data not shown), all the model fit indexes indicated a good fit in men and in women. In the age-group of 25-44 years, opinions on PE in childhood had a significant direct effect (men B = 0.30; women B = 0.39) in competitive sports in youth and exercise in late adolescence a direct effect (men B = 0.15; women B = 0.16) on adulthood LTPA. These two direct effects were adjusted for age in educational group analyses, but the associations did not change and gave only a poorer model fit. The age-adjustment was not applied in the educational group analyses.

In educational group analyses, all the model fit indexes indicated a good fit to the data (men TLI = 1.00, CFI = 1.00, RMSEA = 0.016, WRMR = 0.684; women TLI = 1.00, CFI = 1.00, RMSEA = 0.022, WRMR = 0.799). All the associations were positive between the indicators and the latent variables.

**Figure 1:**
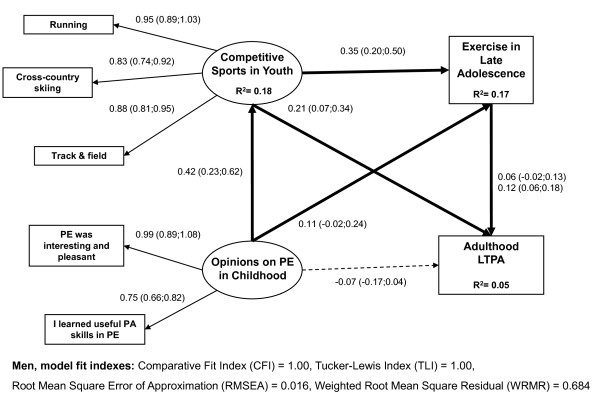
**The effects of adolescence sports and exercise on adulthood leisure-time physical activity in low-educated men**. Multi-group structural equation model with latent variables for the effects of competitive sports in youth, exercise in late adolescence, and opinions on physical education (PE) on adulthood leisure-time physical activity (LTPA) in low-educated men (N = 813), standardized coefficients, 95% confidence intervals, and squared residuals (R2).

**Figure 2:**
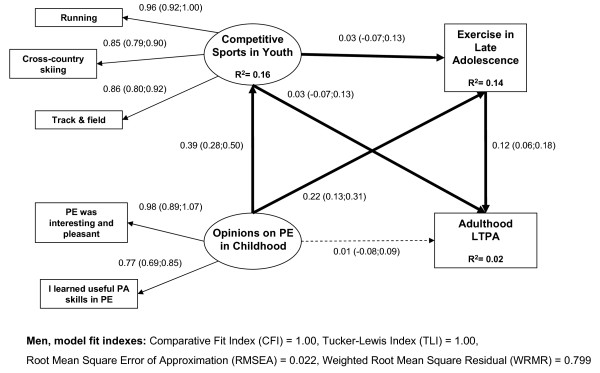
**The effects of adolescence sports and exercise on adulthood leisure-time physical activity in high-educated men**. Multi-group structural equation model with latent variables for the effects of competitive sports in youth, exercise in late adolescence, and opinions on physical education (PE) on adulthood leisure-time physical activity (LTPA) in high-educated men (N = 1674), standardized coefficients, 95% confidence intervals, and squared residuals (R2).

Participation in competitive sports in youth in the low-educated (men B = 0.21; women B = 0.14) and exercise in late adolescence in the high-educated (men B = 0.12; women B = 0.14) had a direct effect on adulthood LTPA (Figure 1 and 2). Positive opinions on PE in childhood have different indirect effects on adulthood LTPA in the low and high-educated participants. In the low-educated, the indirect effect of PE came through participation in competitive sports in adolescence (men B = 0.42 × 0.23 = 0.097; women B = 0.41 × 0.14 = 0.057) whereas in the high-educated, the indirect effect came through leisure-time exercise in late adolescence (men B = 0.026; women B = 0.036). In the high-educated women, opinions on PE in childhood also had an indirect effect on competitive sports in youth and exercise in late adolescence to adulthood LTPA (B = 0.41 × 0.19 × 0.14 = 0.01). The direct and indirect effects in educational groups were largely similar between genders.

Based on the squared residuals (R^2^), opinions on PE in childhood explained nearly 20 percent of the variation in competitive sports in youth in both educational groups among both men and women (Figure 1 and 2). In the low and high-educated men, opinions on PE in childhood and competitive sports in youth explained nearly 20 percent of the variation in exercise in late adolescence. In women, opinions on PE in childhood and competitive sports in youth explained 14 percent of the variation in exercise in late adolescence among the high-educated whereas in the low-educated they explained only 5 percent of the variation. In both educational groups, all the examined youth and adolescence determinants explained only 5 percent or less of the variation in adulthood LTPA among men and women.

## Discussion

Our study is, so far, the first to examine how childhood and adolescent physical activity determines adulthood leisure-time physical activity (LTPA) in low- and high-educated groups. Our findings show educational differences in direct and indirect effects of adolescence sports and exercise and opinions on PE in childhood on adulthood LTPA. Participation in competitive sports in youth had a direct effect on adulthood LTPA only in the low-educated, whereas exercise in late adolescence had a direct effect on adulthood LTPA in the high-educated. In the low-educated, the indirect effect of opinions on PE in childhood came through participation in competitive sports in youth whereas in high-educated the indirect effect came through exercise in late adolescence. The effects were mainly similar between genders.

Participation in intensive endurance sports is shown to predict a high level of physical activity in adulthood [16,19]. Our results were partly similar in the low-educated. Those who had participated in cross-country skiing, running, or track and field in youth did more LTPA in adulthood compared to those who had not participated. The cross-country skiing, running and track and field were chosen since they were the most common competitive sports among the respondents. In the low-educated men and the high-educated women, participation in these competitive sports in youth was also associated with more vigorous exercise in late adolescence. In the high-educated, only vigorous exercise in late adolescence was associated with adulthood LTPA. In our data, however, only a few who had participated in competitive sports in youth participated in these sports also in adulthood. This suggests that although participation in competitive sports might end during the transition from adolescence to adulthood, one might maintain their high level of LTPA from adolescence to adulthood. Our results are in line with a Swedish prospective study [43], where both the breadth of physical activity experiences in adolescence, including aspects such as enjoyment, competition (less in girls) and learning new skills, predicted physical activity in adulthood.

Opinions on PE in childhood had an indirect effect on adulthood leisure-time physical activity. One must remember that in Finland, PE includes only the weekly physical activity curriculum and not physical activity clubs or school-team sports. Our result generally concords with several prospective studies [12,14-17]: a persistent high level of physical activity during school age predicts higher level of LTPA in adulthood. Contradictory findings suggest that high participation in school physical activity might not necessarily predict adulthood LTPA [20,21]. It could be that those who have positive opinions on PE in childhood are more active sports club members and participants in organised sports [12] as compared to those with negative opinions on PE. However, it may as well be that those who competed in certain sports have more positive opinions on PE in childhood and they also did more leisure-time exercise compared to other individuals.

Importantly, the indirect effect of opinions on PE in childhood was different between educational groups. In the low-educated, those who had positive opinions on PE in childhood and had participated in competitive sports were also physically active during leisure-time in adulthood. In the high-educated, the effect of positive PE opinions in childhood on adulthood LTPA came through adolescent leisure-time exercise. Educational differences in physical activity are unlikely to develop during elementary school whereas the transition after school might have a more important role in shaping adulthood physical activity [44]. A career requiring a high-education might strengthen health consciousness [45] and this associates strongly to physical activity in adolescence [23] and adulthood [46]. High-educated individuals might therefore engage in leisure-time exercise due to its health benefits. Low-educated individuals, on the other hand, might be physically active due to a socio-cultural team spirit or to gain a different kind of success to educational success.

The effects adolescence sports and exercise as well opinions on PE in childhood on adulthood LTPA were mainly similar among women and men. In both genders, participating in competitive sports in youth or exercising in late adolescence predicted higher LTPA in adulthood. This suggests the educational pathways of physical activity from adolescence to adulthood are mainly similar between genders.

## Limitations and strengths

We applied structural equation modelling as statistical method in our analyses. We reported all the results using commonly accepted practice, but we have to remember that data was cross-sectional in nature and although we speak of effect no causal assumptions can be made. Although it would be optimal to utilize longitudinal data to examine the effects of adolescence factors to adulthood factors, retrospectively data collected in adulthood enables us to determine a chronological order between adolescent and adulthood factors. Self-reported measures are a common way to assess physical activity in large populations, but are known to be influenced by a recall bias [47]: average recall bias amounts to half a unit in a four-point scale, while the elderly are more likely to overestimate their physical activity level in the distant past. For some of the participants there could be more than 45 years of recall time, this could affect their responses on adolescence sports and exercise. In addition, the retrospective questions on childhood and adolescence physical activity have not been validated before of this study. Current perceptions on physical activity might also affect respondents' answers on adolescence sports and exercise. For example it could also be that respondents who are physically active in adulthood might recall and report more favourable attitudes toward PE in childhood.

Results from the retrospective physical activity measurements should therefore be interpreted with caution. Our 12-month recall on adulthood LTPA has been validated against maximal oxygen uptake (VO_2 _max) [36]. We were also able to calculate relative energy expenditure (METh/wk) which is not often done in population-based studies. However, one must also remember that we focused only on total LTPA including commuting PA, conditioning PA, and household chores as well as other daily PA. We did not include occupational PA which has been shown to contribute to LTPA [27].

The different recall time in retrospective questions between the participants from younger and older birth cohorts might have influenced their answers. Younger participants might remember more accurately their physical activity history compared to older participants. In addition, the Finnish school system has varied between older and younger age-cohorts. Some of the older age-cohorts were also not able to go the senior high school due to poor living conditions and long distances to the nearest school after several war situations in Finland during 1940s. In addition, one must remember that PE was quite different among participants from older birth cohort than among participants from younger birth cohorts. The age-group analyses in both genders, however, indicated that age had only a small effect on the associations between the opinions on PE at school and competitive sports in youth. A similar effect was found between exercise in late adolescence and adulthood LTPA. The associations in the educational group models did not markedly change after adjusting for age. It might also be that the high-educated based their answers relating to physical education and physical activity on their time in high school whereas the low-educated on their time in elementary school.

Non-response might have influenced our results at two points of the analysis. Compared to the original population sample (N = 13436), those who did not participate in the National FINRISK 2002-study were more likely to be young and low-educated. In addition, those who did not participate in the physical activity sub-study and did not complete the 12-month recall on adulthood LTPA were more often men, older, low-educated, and physically inactive during their leisure-time. Although we cannot be certain of the physical activity level of the young non-respondent, the educational differences in LTPA would be even wider if non-respondents could be taken into account.

## Conclusions

Physical inactivity has become a public health problem and clear socioeconomic differences in physical inactivity exist in most of the European countries [3,9]. However, hardly any studies have examined the determinants of LTPA in different educational groups among adults. Based on our study, the pathways of physical activity from childhood to adulthood LTPA may be different depending on the educational requirement of the pursued career. Competitive sports in youth predicted adulthood LTPA among the low-educated and exercise in late adolescence among the high-educated. Opinions on PE had different indirect effects depending on education. Our study assessed the determinants of LTPA which could be applied to promote physical activity in low-educated individuals. Since participation in competitive sport in youth seemed to have positive effects on later physical activity among low-educated individuals, further promotion of competitive sports among low-educated individuals could have positive effects on adopting life-long physically active life-style. Further prospective studies are, however, needed to confirm our results.

## Competing interests

The authors declare that they have no competing interests.

## Authors' contributions

TEM has substantially contributed to conception and design of the study, to analysis and interpretation of data, involved in drafting the manuscript, acted as the corresponding author, has read and approved the final manuscript. KB has substantially contributed to conception and design of the study, to analysis and interpretation of data, involved in drafting the manuscript, has read and approved the final manuscript. THT has substantially contributed to conception and design of the study, to analysis and interpretation of data, involved in drafting the manuscript, has read and approved the final manuscript. OR has substantially contributed to conception and design of the study, to analysis and interpretation of data, involved in drafting the manuscript, has read and approved the final manuscript. TL has substantially contributed to conception and design of the study, to analysis and interpretation of data, involved in drafting the manuscript, has read and approved the final manuscript. RP has substantially contributed to conception and design of the study, to analysis and interpretation of data, involved in drafting the manuscript, has read and approved the final manuscript.
